# Distinct neural signatures of schizotypy and psychopathy during visual word‐nonword recognition

**DOI:** 10.1002/hbm.25872

**Published:** 2022-04-18

**Authors:** Martina Vanova, Luke Aldridge‐Waddon, Ray Norbury, Ben Jennings, Ignazio Puzzo, Veena Kumari

**Affiliations:** ^1^ Division of Psychology, Department of Life Sciences, & Centre for Cognitive Neuroscience, College of Health, Medicine and Life Sciences Brunel University London London UK

**Keywords:** lexical decision, motor impulsivity, psychopathy, reading, schizotypy

## Abstract

Previous behavioural data indicate lower word‐nonword recognition accuracy in association with a high level of positive schizotypy, psychopathy, or motor impulsivity traits, each with some unique contribution, in the general population. This study aimed to examine the neural underpinnings of these associations using functional magnetic resonance imaging (fMRI) in a volunteer sample. Twenty‐two healthy English‐speaking adults completed self‐report measures of schizotypy (Oxford‐Liverpool Inventory of Feelings and Experiences [O‐LIFE]), psychopathy (Triarchic Psychopathy Measure [TriPM]), and impulsivity (Barratt Impulsiveness Scale [BIS‐11]) and underwent whole‐brain fMRI while performing a lexical decision task (LDT) featuring high and low‐frequency words, real nonwords, and pseudohomophones. Higher positive schizotypy (Unusual Experiences) was associated with lower cerebellum activity during identification of low‐frequency words (over real nonwords). Higher Boldness (fearless dominance) and Meanness (callous aggression) facets of psychopathy were associated with lower striatal and posterior cingulate activity when identifying nonwords over words. Higher Motor Impulsivity was associated with lower activity in the fusiform (bilaterally), inferior frontal (right‐sided), and temporal gyri (bilaterally) across all stimuli‐types over resting baseline. Positive schizotypy, psychopathy, and impulsivity traits influence word‐nonword recognition through distinct neurocognitive mechanisms. Positive schizotypy and psychopathy appear to influence LDT performance through brain areas that play only a supportive (cerebellum) or indirect role in reading‐related skills. The negative association between Motor Impulsivity and activations typically found for phonological processing and automatic word identification indicates a reduced bilateral integration of the meaning and sound of mental word representations, and inability to select the appropriate outputs, in impulsive individuals.

## Graphical abstract

Positive schizotypy and Motor Impulsivity influence word‐nonword recognition through distinct neurocognitive mechanisms. Higher Motor Impulsivity was associated with lower activity in brain areas typically involved in phonological processing and automatic word identification, namely the fusiform gyrus bilaterally, right inferior frontal gyrus, and temporal gyri bilaterally, during word‐nonword recognition. Positive schizotypy was associated with activity modulation in the cerebellum, which is considered to play a supportive role in phonological and semantic processing.

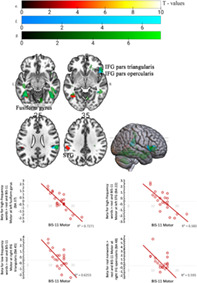

## 1 INTRODUCTION

Reading as a process requires decoding written symbols into verbal information (phonological processing), word identification, and subsequent comprehension of its meaning (Pollatsek et al., 2000). Brain areas associated with phonological processing (inferior frontal gyrus [IFG], fusiform gyrus) and comprehension (basal temporal areas) show activation during correct recognition of words (Fiez, 2001). Word‐nonword recognition, as assessed by a lexical decision task (LDT), is reported to be accompanied by activation of the fusiform gyrus, anterior cingulate, IFG, and the middle and posterior superior temporal gyrus (STG) (Kiehl et al., 2004), with the occipital areas, alongside the collateral sulcus and fusiform gyrus, showing stronger activation for words than nonwords (Fiebach et al., 2002). Broca's areas (IFG), known to be involved in the phonological part of the language, is strongly activated during nonword recognition whereas word recognition involves more temporal areas involved in semantic processing (Paz‐Alonso et al., 2018; Wimmer et al., 2010).

There is also evidence of left inferior frontal (Broca's areas) activity correlating with task difficulty as determined by word frequency (Carreiras et al., 2006; Fiebach et al., 2002; Liu et al., 2004). Low‐frequency words elicit stronger activity than high‐frequency words in left IFG (pars opercularis), left inferior parietal lobule (implicated in orthography‐phonology integration), left middle temporal gyrus (implicated in semantic processing) (Newman & Joanisse, 2011) and also in anterior cingulate and supplemental motor areas (Carreiras et al., 2006); high‐frequency words show relatively stronger activity in the cingulate and inferior parietal regions (Nakic et al., 2006). Pseudohomophones (nonwords resembling or sounding like words) produce significantly more activation in areas involved in phonological processing (left IFG and precentral gyrus) and semantic processing (pars triangularis IFG) than real nonwords (Edwards et al., 2005; Newman & Joanisse, 2011). Real nonwords produce stronger activation than pseudohomophones, especially in occipitotemporal regions, IFL, and precentral areas (Wimmer et al., 2010). In general, lexical stimuli requiring a higher level of phonological processing, such as low‐frequency (unfamiliar) words and nonwords, activate the IFG and precentral gyrus, whereas high‐frequency words which do not require this level of processing activate semantic areas in the temporal lobe.

In addition to task‐related factors, the pattern of brain activation may vary in relation to individual differences, for example, certain psychopathology‐related dimensions that negatively influence LDT performance (Harmon‐Jones et al., 1997; Heritage & Benning, 2013; Lorenz & Newman, 2002; Tan et al., 2016). Our recent study (Vanova et al., 2022) showed negative associations between LDT performance (accuracy) and Unusual Experiences (positive schizotypy) subscale of the Oxford‐Liverpool Inventory of Feelings and Experiences (O‐LIFE; Mason & Claridge, 2006), Boldness (fearless dominance), and Meanness (callous aggression) facets of psychopathy (Triarchic Psychopathy Measure [TriPM]; Patrick et al., 2009), as well as Motor Impulsivity (Barratt Impulsiveness Scale [BIS‐11]; Patton et al., 1995) in a healthy volunteer sample. Importantly, we found that each of these traits explained some amount of unique variance in performance. Specifically, Meanness accounted for 12%, Boldness 4.8%, and Unusual Experiences accounted for 4.4% of the total variance in word‐nonword recognition accuracy. Motor Impulsivity explained 30% of the variance in low‐frequency word recognition accuracy but only in non‐native English speakers, who as a group had shown lower word‐nonword recognition accuracy than native speakers. These observations raise the possibility that associations between LDT performance and different psychopathology‐related traits, namely positive schizotypy, psychopathy, and impulsivity, may be mediated, at least partially, by different cognitive processes and represented at the brain level by different brain activation patterns.

The aims of this study were therefore to examine the neural correlates of the associations between LDT performance and the traits of positive schizotypy, psychopathy, and impulsivity using whole‐brain functional magnetic resonance imaging (fMRI) in a healthy volunteer sample. The LDT task utilised for this investigation featured high and low‐frequency words, real nonwords, and pseudohomophones. We expected that brain areas that are known to be active in phonological processing depending on the degree of phonological processing required (i.e., left IFG, left insula, precentral gyrus bilaterally) will show the strongest activation with correct identification of pseudohomophones and real nonwords, and lowest activation during the high‐frequency words, with low‐frequency words showing the intermediate level of activity. We tentatively hypothesised that higher levels of positive schizotypy (Unusual Experiences), psychopathy (Meanness, Boldness), and Motor Impulsivity will correlate with lower activation in areas associated with phonological processing when identifying low‐frequency words, real nonwords, and pseudohomophones in order of increasing involvement of phonological processing.

Furthermore, we expected to see some distinct neural correlates for positive schizotypy, psychopathy, and impulsivity along with relatively stronger associations with brain activity in some areas for Meanness and/or Motor Impulsivity, given that these traits had explained some unique but varying amount of variance in LDT performance in our previous study (Vanova et al., 2022).

## 2 METHODS

### 2.1 Participants and design

Twenty‐two healthy right‐handed adults (>18 years; 15 women, see Table S1) were recruited via the university network. All participants were required to have a sufficient written and verbal command of the English language (meeting university entry requirements), normal or corrected‐to‐normal vision and hearing, no history or current diagnosis of any psychiatric or neurological illness, and no serious offence history as self‐reported. They were prescreened for any MRI contraindications. All participants were assessed on one occasion in the fMRI scanner and completed self‐report questionnaires online within two days of the fMRI session.

This research was approved by the College of Health, Medicine and Life Sciences Research Ethics Committee, Brunel University London (Reference: 16789‐MHR‐May/2019–19042‐2). All participants gave written informed consent after the study procedures had been explained to them.

### 2.2 Self‐report questionnaires

Schizotypy was assessed using the O‐LIFE (150 items; subscales: Unusual Experiences, Cognitive Disorganisation, Introvertive Anhedonia, Impulsive Nonconformity) (Mason & Claridge, 2006). Psychopathy was assessed using the TriPM (58 items; subscales: Boldness, Meanness, Disinhibition) (Patrick et al., 2009). Impulsivity was assessed using the BIS‐11 (30 items; subscales: Attention, Cognitive Instability, Motor, Perseverance, Self‐Control, Cognitive Complexity) (Patton et al., 1995). All questionnaires were administered online using Qualtrics^XM^ (Qualtrics LLC, 2005).

### 2.3 fMRI: Paradigm and procedure

The LDT was administered using Presentation Software (version 21.1) (Neurobehavioral Systems Inc., 2018). Participants were presented with 120 stimuli (60 words, 60 nonwords), 5–6 letters long, in three blocks of 40 stimuli each, counterbalanced for frequency, and pseudo‐randomised order. Each trial was 700 ms long (500 ms stimulus presentation, and 100 ms interstimulus intervals, one at the beginning and one at the end) and preceded by a random 1000 ms to 5000 ms jitter (average 3000 ms). A 15‐second blank screen was presented between the three blocks of stimuli. The overall experiment duration was 474 s.

The word list consisted of 30 high‐frequency word lemmas, 300–306 occurrences per million words and 30 low‐frequency word lemmas, 10–11/million, all retrieved from the British National Corpus (Leech et al., 2001). The nonword list included 30 real nonwords and 30 pseudohomophones from the ARC Database (Rastle et al., 2002). The nonword list was counterbalanced in the summed frequency of nonword neighbours, which is an indicator of similarity with other nonwords (high frequency: 300–700/million; low frequency: 0–10/million). The neighbourhood size of each nonword and pseudohomophone was one. This refers to the number of words that can be derived by changing one letter while preserving the position of the other letters. All nonword stimuli were orthographically legal – consisting of combinations of letters proper to the English language.

A four‐button MRI compatible response box (Lumitouch, Photon Control Inc., Baxter, Canada) was used to record responses.

#### 2.3.1 fMRI: Data acquisition

The data were acquired on 3 Tesla Siemens TIM Trio whole‐body MRI scanner (Siemens Medical Solutions, Erlangen, Germany) at the Combined Universities Brain Imaging Centre (CUBIC), Royal Holloway University London, fitted with a 32‐channel head coil. The functional images were acquired in one run using the following pulse sequence: TR = 2000 ms, TE = 30.6 ms, 50 interleaved slices, voxel size = 2 × 2 × 3 mm, flip angle = 78°, field of view = 192 mm, base resolution = 96, 96 × 96 matrix. Time correction was based on the middle slice and realignment reference volume was the first volume. A total of 242 volumes were obtained during the experiment. High‐resolution T1‐weighted images were acquired during the same session with the following parameters: TR = 2300 ms, TE = 2.9 ms, 192 images of 1 × 1 × 1 mm voxel size, flip angle = 9°, field of view = 256 mm, base resolution = 256, matrix 256 × 256.

### 2.4 Data analysis

#### 2.4.1 Behavioural data

The data were analysed using IBM SPSS Statistics, Version 26.0 (IBM Corp., 2019), with alpha level for significance testing set at <0.05, unless stated otherwise. The skewness of all LDT performance and self‐report variables was checked and found to be within the acceptable range (Field, 2009), except for the correct low‐frequency words and correct real nonwords which were mildly skewed (max. *z* = −2.271). No corrections were applied. Performance accuracy and RTs were analysed using a repeated‐measure analysis of variance (ANOVA) with Stimulus‐Type (high‐frequency words, low‐frequency words, real nonwords, pseudohomophones) as a within‐subject variable. Prior to running these analyses, we had conducted mixed model ANOVAs on these data with stimulus‐type as a within‐subject variable, and Sex (males, females) or Language (native vs. nonnative speakers) as the between‐subject factors (Sex and Language examined in separate ANOVAs, given the relatively small sample size). As we observed no significant main effect of Sex or Language, and no interaction involving these factors (all *p* > .27), all reported results are from ANOVAs with repeated measures on Stimulus‐Type. The Greenhouse–Geisser correction was applied to all repeated measures statistics where Mauchly's Test indicated a significant violation of sphericity (*p* < .05). Post‐hoc mean comparisons were conducted to probe significant effects as required. Effect sizes were calculated as partial eta squared (*η* ^2^_*p*_) and interpreted as follows: *η* ^2^_*p*_ ≥ .01 to <.06 (small), *η* ^2^_*p*_ ≥ .06 to <.14 (medium), *η* ^2^_*p*_ ≥ .14 (large) (Cohen, 1992). Cohen's *d* values were interpreted as follows: ≥.2 to <.5 (small standardised effect size), ≥.5 to <.8 (medium), and ≥.8 (large) (Cohen, 1992). Pearson correlation coefficient with two‐tailed significance was used to examine hypothesised LDT performance‐traits associations.

#### 2.4.2 fMRI data

SPM12 toolbox (Friston et al., 2007) for MATLAB R2020a (MATLAB, 2020) was used for data pre‐processing and analysis, and the MRIcroGL (Rorden & Brett, 2000) for graphic visualisation. At the beginning of the pre‐processing, the anterior commissure was manually set as an origin for the structural and all functional images. All functional images were realigned and co‐registered with the corresponding structural images for each participant. The resulting images were normalised to the Montreal Neurological Institute (MNI) space with 2 × 2 × 2 mm voxel resolution for functional images, and forward deformations field. The transformation parameters were obtained from the segmentation of structural images. The normalised images were then smoothed with full width at a half‐maximum (FWHM) Gaussian smoothing kernel of 10 mm.

The smoothed images were then subjected to a two‐level analysis. At the first level, we performed a random‐effect analysis of participant‐specific contrast activations (i.e., four stimuli‐types compared to the implicit baseline‐resting condition, and one another). At the second level, we identified task‐related neural activations using one‐sample *t* tests across the entire sample (height threshold *p* < .001; family‐wise error [FWE] corrected for multiple comparisons at the cluster level *p* < .05). The relationships of psychopathology‐related traits (O‐LIFE Unusual Experiences, TriPM Meanness and Boldness, and BIS‐11 Motor Impulsivity) with neural activity across the whole brain for each contrast were then examined using a regression model within SPM12 with questionnaire scores entered as a covariate (height threshold *p* < .001; cluster‐corrected *p* ≤ .05). Next, the participant‐specific activation values were extracted (from one‐sample *t* tests including all participants for relevant contrasts) from the regions (peak voxel) that had shown an association with positive schizotypy, psychopathy, or Motor Impulsivity in SPM regression analyses (see Section 3) and then examined (within the SPSS) using correlational analyses to find out whether the observed personality‐brain associations were, or not, related to individual differences in performance.

## 3 RESULTS

### 3.1 Sample characteristics

Participants' age range was 19–42 years (*M* = 24.14; SD = 5.40), and education at undergraduate (50%) or postgraduate level (50%). Males and females did not differ in age, ethnicity, or self‐report measures (all *p* values >.05). There were equal numbers of native and non‐native speakers (11 per group). Full sample characteristics are provided in Table S1.

### 3.2 LDT performance

#### 3.2.1 Accuracy

There was a significant main effect of Stimulus‐Type with a large effect size (*F*(1.75,36.75) = 28.854, *p* < .001, *η* ^2^_*p*_ = .579). Participants identified significantly more high than low‐frequency words (*t*(21) = 4.945, *p* < .001, Cohen's *d* = 1.414), more low‐frequency words than pseudohomophones (*t*(21) = 4.622, *p* < .001, Cohen's *d* = .766), and more real nonwords than pseudohomophones (*t*(21) = 3.775, *p* = .001, Cohen's *d* = .769), meaning that the pseudohomophone effect was present (Table 1).

**TABLE 1:** Descriptive statistics for LDT performance and psychopathology‐related traits (*n* = 22)

Measure	Mean (SD)	Min.	Max.	Max. possible
LDT performance				
Correct high‐frequency words	29.227 (.92)	27	30	30
Correct low‐frequency words	27.500 (1.47)	24	29	30
Correct real nonwords	25.546 (3.31)	17	30	30
Correct pseudohomophones	23.682 (3.79)	16	30	30
Incorrect high‐frequency words (*n* = 18)	.727 (.83)	0	2	30
Incorrect low‐frequency words (*n* = 18)	2.273 (1.35)	1	6	30
Incorrect real nonwords (*n* = 18)	3.954 (3.23)	0	13	30
Incorrect pseudohomophones (*n* = 18)	5.954 (3.81)	0	14	30
Missed (*n* = 10)	1.136 (2.08)	0	9	120
Correct high‐frequency words RT (ms)	565.718 (85.84)	420	724	2000
Correct low‐frequency words RT (ms)	648.584 (110.24)	460	901	2000
Correct real nonwords RT (ms)	723.149 (135.90)	539	1059	2000
Correct pseudohomophones RT (ms)	740.494 (140.41)	540	989	2000
Psychopathology traits				
O‐LIFE Unusual Experiences	8.910 (3.915)	0	17	30
TriPM Boldness	29.770 (7.118)	15	45	76
TriPM Meanness	12.590 (6.493)	3	25	76
BIS‐11 Motor Impulsivity	14.500 (3.635)	9	24	28

Abbreviations: BIS‐11, Barratt Impulsiveness Scale; O‐LIFE, Oxford‐Liverpool Inventory of Feelings and Experiences; RT, reaction time; TriPM, Triarchic Psychopathy Measure.

#### 3.2.2 RTs

There was a significant main effect of Stimulus‐Type with a large effect size (*F*(3,63) = 78.326, *p* < .001, *η* ^2^_*p*_ = .789). A follow‐up analysis showed that reaction times (RTs) pattern was identical to that seen in accuracy. Participants were significantly faster when identifying high than low‐frequency words (*t*(21) = 8.342, *p* < .001, Cohen's *d* = .839), low‐frequency words than pseudohomophones (*t*(21) = 6.444, *p* < .001, Cohen's *d* = .728), but the RT differences between pseudohomophones and real nonwords was not significant (*t*(21) = 1.897, *p* = .072, Cohen'*s d* = .126) (Table 1).

#### 3.2.3 LDT performance: Speed‐accuracy trade‐off

Longer RTs correlated positively with recognition accuracy of high‐frequency words (*r* = .470, *p* = .027). No further correlations were found in the entire sample, or when explored separately in native and non‐native language groups.

#### 3.2.4 Relationship between LDT performance and psychopathology‐related traits

No correlations between psychopathology‐related traits and LDT performance reached formal significance, though LDT accuracy was associated with positive schizotypy (Unusual Experiences), and Motor Impulsivity in the expected direction (i.e., negatively; see Table 2).

**TABLE 2:** Correlations between LDT accuracy and psychopathology‐related measures

	Overall performance	Correct words high‐frequency	Correct words low‐frequency	Correct real nonwords	Correct pseudohomophones
Measure	*r* (*p*)	*r* (*p*)	*r* (*p*)	*r* (*p*)	*r* (*p*)
O‐LIFE Unusual Experiences	−.224 (.316)	−.126 (.577)	−.331 (.133)	−.272 (.221)	−.063 (.780)
TriPM Boldness	.099 (.662)	.153 (.496)	−.134 (.552)	.046 (.839)	.178 (.429)
TriPM Meanness	.223 (.318)	.350 (.110)	.052 (.817)	.097 (.666)	.268 (.228)
BIS‐11 Motor Impulsivity	−.365 (.095)	−.263 (.237)	−.414 (.056)	−.400 (.065)	−.175 (.437)

Abbreviations: BIS‐11, Barratt Impulsiveness Scale; O‐LIFE, Oxford‐Liverpool Inventory of Feelings and Experiences; TriPM, Triarchic Psychopathy Measure.

### 3.3 Functional magnetic resonance imaging

#### 3.3.1 Task‐related activations

**FIGURE 1:**
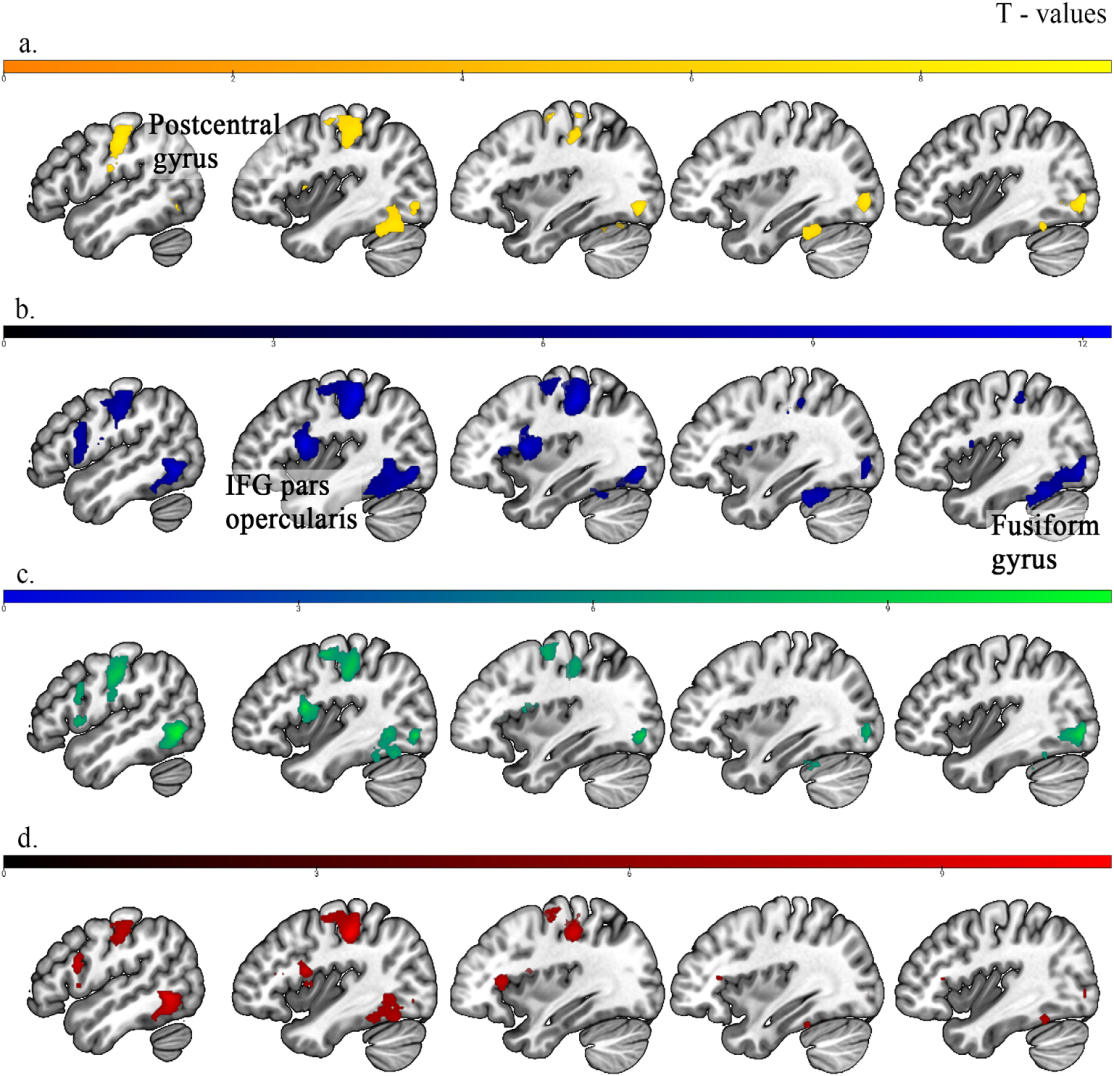
Areas of higher brain activity over resting baseline for: (a) high‐frequency words (yellow), (b) low‐frequency words (blue), (c) real nonwords (green), and (d) pseudohomophones (red) (*n* = 22) at *x* = −50, −40, −35, 35, 40 (sagittal view). L, left; R, right

Brain activation changes associated with all task contrasts are detailed in Table S2. As can be seen in Figure 1, the IFG—pars opercularis (bilaterally), inferior occipital gyrus (bilaterally), fusiform gyrus (bilaterally), postcentral gyrus (left), and insula (left) were activated for all Stimulus‐Types compared to rest (Figure 1). For high‐frequency words, the main activated areas were the right inferior occipital gyrus, left postcentral and fusiform gyri. For low‐frequency words, large clusters of activation were found in the left hemisphere at Rolandic operculum, postcentral, and inferior occipital gyri. For pseudohomophones, left‐sided activity, mainly in the postcentral, inferior temporal, and fusiform gyri, and in the Rolandic operculum was found. For real nonwords, activation was present in the left postcentral, inferior temporal, and Rolandic operculum, and the right inferior occipital and fusiform gyri. The main activated region for high‐frequency words, in comparison to nonwords, was in the left angular gyrus. The low‐frequency words recognition over high‐frequency words showed the strongest activations in the IFG—pars triangularis, and in the inferior temporal gyrus.

#### 3.3.2 Relationship between brain activations and psychopathology‐related traits

**FIGURE 2:**
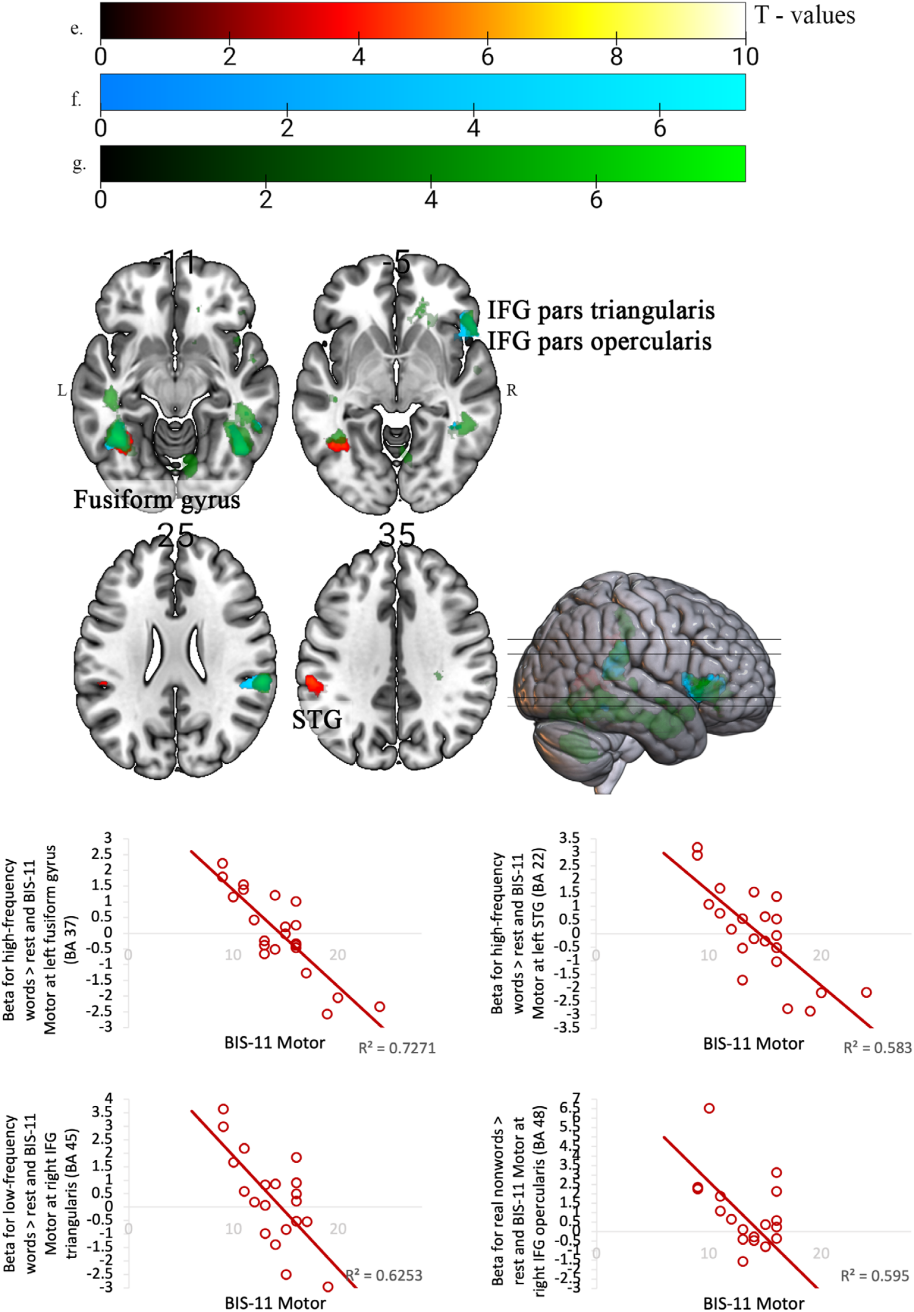
Areas of brain activity negatively associated with Motor Impulsivity during the correct identification of: (a) high‐frequency (left fusiform gyrus and left STG), (b) low‐frequency words (fusiform gyrus bilaterally and right IFG pars triangularis), and (h) real nonwords (fusiform gyrus bilaterally, right IFG pars opercularis). L, left; R, right. Images for z‐coordinates (axial plane)

**FIGURE 3:**
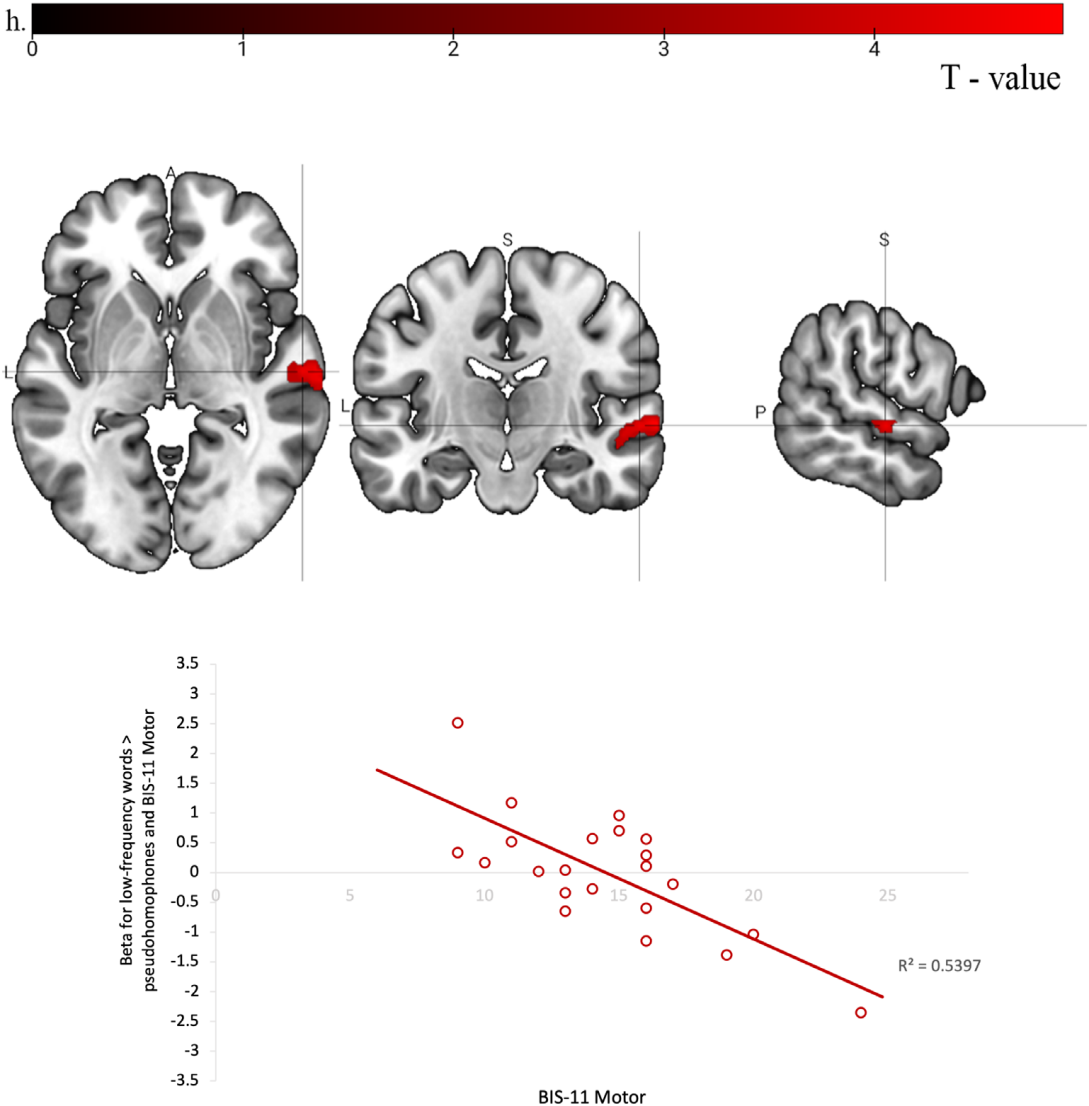
Areas of brain activity (right STG; peak MNI coordinates: *x* = 60, *y* = −16, *z* = −2) negatively associated with Motor Impulsivity to the correct identification of: (a) low‐frequency words over pseudohomophones. A, anterior; L, left; P, posterior; S, superior

All brain areas showing a relationship with one or more psychopathology‐related traits are described in Table 3. Specifically, lower Motor Impulsivity was associated with higher activation in the fusiform gyrus bilaterally for correctly identified high and low‐frequency words, and real nonwords (all over rest) (Figure 2). Lower Motor Impulsivity was also associated with higher activation in the right STG when identifying low‐frequency words over pseudohomophones (Figure 3). Higher positive schizotypy (O‐LIFE ‐ Unusual Experiences) scores were associated with lower activation in the left cerebellum when identifying low‐frequency words over real nonwords. Higher Meanness was associated with higher activity, especially in the left caudate nucleus when identifying high‐frequency words over real nonwords. Similarly, higher Boldness was associated with a small cluster of higher activity in the right posterior cingulate when identifying low‐frequency words over pseudohomophones. Overall, Motor Impulsivity was most frequently and strongly associated with task‐related activations.

**TABLE 3:** Relationship (negative associations) between task‐related activations and psychopathology‐related traits (height threshold *p* < .001 uncorrected)

Contrast/psychopathology trait			Cluster level			Peak level		MNI coordinates		
Area name	BA	Side	*P*_FWE_	*K*_*E*_	*P*_uncor._	*P*_uncor._	*T*	x y z (mm)		
**O‐LIFE Unusual Experiences**										
Correct low‐frequency words > correct real nonwords										
Cerebellum		Left	.004	355	<.001	<.001	6.03	−6	−76	−28
						<.001	5.28	−2	−72	−38
**TriPM Meanness**										
Correct real nonwords > high‐frequency words										
Ventral diencephalon	25	Left	.019	281	.002	<.001	6.66	−6	−4	−12
Caudate nucleus						<.001	5.51	−2	8	−10
		Right				<.001	4.64	4	−2	−12
**TriPM Boldness**										
Correct pseudohomophones > low‐frequency words										
Posterior cingulate		Right	.037	234	.005	<.001	5.97	12	−38	24
						<.001	4.71	22	−40	30
						<.001	4.16	28	−48	24
**BIS‐11 Motor Impulsivity**										
Correct high‐frequency words > rest										
Fusiform gyrus	37	Left	<.001	866	<.001	<.001	10.10	−44	−54	2
						<.001	5.98	−38	−52	−16
						<.001	5.52	−34	−58	−8
Superior temporal gyrus	22	Left	.002	462	<.001	<.001	6.11	−64	−38	12
	42					<.001	5.87	−48	−40	12
						<.001	4.97	−56	−42	16
Inferior frontal opercularis	47	Right	.016	289	.002	<.001	5.51	48	26	−2
Inferior frontal triangularis	45					<.001	4.34	50	34	8
Insula	48					<.001	4.01	42	16	2
Correct low‐frequency words > rest										
Fusiform gyrus	37	Left	.001	477	<.001	<.001	7.07	−40	−52	−16
						<.001	4.15	−32	−32	−20
Fusiform gyrus	42	Right	.001	449	<.001	<.001	6.99	60	−34	24
Superior temporal gyrus						<.001	5.79	58	−42	12
Inferior frontal triangularis	45	Right	<.001	615	<.001	<.001	6.91	48	28	4
						<.001	6.45	50	22	−2
						<.001	6.13	50	36	4
Inferior temporal gyrus	37	Right	.011	274	.001	<.001	5.16	44	−60	−12
						<.001	4.39	42	−48	−12
	20					<.001	4.27	56	−44	−12
Correct real nonwords > rest										
Inferior temporal gyrus	36	Right	<.001	2076	<.001	<.001	8.17	40	−4	−28
	20					<.001	6.58	46	−60	−12
						<.001	6.19	44	−16	−20
Fusiform gyrus	37	Left	<.001	1178	<.001	<.001	7.78	−42	−50	−14
						<.001	7.38	−42	−54	2
						<.001	5.51	−46	−28	−10
Cerebellum		Right	<.001	938	<.001	<.001	6.78	18	−60	−40
						<.001	5.62	10	−64	−36
		Left				<.001	4.84	−12	−64	−42
Inferior frontal triangularis	47	Right	.001	438	<.001	<.001	6.32	50	28	0
Inferior frontal opercularis	48					<.001	5.52	58	16	8
	45					<.001	5.05	58	32	4
Inferior frontal triangularis	47	Right	.001	416	<.001	<.001	6.24	32	36	8
						<.001	6.21	26	26	0
						<.001	4.83	20	34	2
Superior temporal gyrus	48	Right	.001	460	<.001	<.001	6.03	60	−34	24
Middle temporal gyrus	42					<.001	5.18	58	−42	12
	21					<.001	4.30	58	−22	0
Postcentral gyrus	3	Right	.019	241	.002	<.001	5.31	26	−30	42
						<.001	4.79	22	−32	52
						<.001	4.55	34	−28	42
Cerebellum		Right	.029	217	.003	<.001	4.97	14	−70	−12
						<.001	4.09	4	−60	0
Cerebellar vermis						.001	3.93	−2	−76	−14
Correct low‐frequency words > correct pseudohomophones										
Superior temporal gyrus	22	Right	.042	225	.005	<.001	5.45	68	−14	0
						<.001	4.67	60	−16	−2
Middle temporal gyrus	20					<.001	4.50	50	−12	−14
Correct real nonwords > correct pseudohomophones										
Cerebellum		Left	.018	335	.003	<.001	4.64	−24	−64	−16
						<.001	4.60	−8	−62	−4
Cerebellar vermis						<.001	4.46	0	−70	−14

Abbreviations: BA, Brodmann area; BIS‐11, Barratt Impulsiveness Scale; FWE, Family‐wise error; MNI, Montreal Neurological Institute coordinate system; O‐LIFE, Oxford‐Liverpool Inventory of Feelings and Experiences; TriPM, Triarchic Psychopathy Measure; uncor., uncorrected.

#### 3.3.3 Relationship between participant‐specific brain activation values, psychopathology traits, and LDT performance

No significant correlations were found between participant‐specific brain activation values for clusters that showed a positive/negative association with one or the other traits and LDT performance (Table S3) except a trend‐level positive correlation between real nonword accuracy (*r* = .393, *p* = .071) and real nonwords (over rest) related activation in the inferior temporal gyrus that was found to be associated with lower Motor Impulsivity.

## 4 DISCUSSION

Higher positive schizotypy (Unusual Experiences) was associated with lower activity in the left cerebellum when recognising low‐frequency words over real nonwords. Higher Boldness was associated with higher activity for low‐frequency words over pseudohomophones in the right posterior cingulate. The only association for higher Meanness reflected higher activation in the ventral diencephalon bilaterally. Motor Impulsivity was the strongest predictor of lower activity, mainly in the fusiform gyrus bilaterally, right IFG, and temporal gyri bilaterally, across all stimuli‐types.

Prior to discussing these associations, we comment on the hypothesised neural networks of LDT activated at the group level.

### 4.1 Task‐related activations

As hypothesised, pars opercularis of the IFG bilaterally, left postcentral gyrus, left insula, and as reported in previous studies (Fiebach et al., 2002; Kiehl et al., 2004), the inferior occipital gyrus, and fusiform gyrus bilaterally were the regions showing task‐related activations. However, the activation was stronger in some areas when identifying high or low‐frequency words over both types of nonwords. Specifically, for high‐frequency words, contrasted with pseudohomophones, the left angular gyrus bilaterally and right precuneus were strongly activated and the left angular gyrus solely when compared to real nonwords. The angular gyrus is crucial for processing whole words and extracting their meanings based on their orthographic properties (Horwitz et al., 1998; Segal & Petrides, 2013). The angular gyrus is also functionally connected with Wernicke's area, facilitating orthographic to phonological processing of words (Pugh et al., 2000). The right precuneus is important in self‐consciousness, self‐awareness, and the theory of mind (Cavanna & Trimble, 2006; Schiffer et al., 2013). However, in reading, together with the angular gyrus, it is involved in context comprehension and coherence (Moss et al., 2011). Thus, the precuneus can act as internal monitoring of the lexical representation meanings previously facilitated by the angular gyrus.

As hypothesised, when identifying low‐frequency over high‐frequency words, greater activity was predominantly in the pars triangularis of the left IFG and the left inferior temporal gyrus to fusiform gyrus, the areas involved in phonological processing (Binder et al., 2006; Dietz et al., 2005; MacSweeney et al., 2009). The fusiform gyrus is more active during quick and easy word recognition in skilled readers in comparison to dyslexics (McCandliss et al., 2003). Therefore, it facilitates a quick translation between the visual word and its sound and meaning (Devlin et al., 2006) by storing and extracting visual and sound patterns for quick recognition (Kronbichler et al., 2004). The pars triangularis of the left IFG was previously found to be active in low but not in high‐frequency word identification or pseudohomophones, facilitating the selection from among competing lexical representations (Fiebach et al., 2002). This area is more active in semantic selection than in phonological processing (Liuzzi et al., 2017; Mechelli et al., 2005). Therefore, the pars triangularis is active later, when the individual is deciding whether the word is identical to any word in their mental vocabulary. The left fusiform gyrus may help to translate the letters quickly and accurately into sounds forming the final words and then in the left pars triangularis is the final representation compared to the existing knowledge.

Low‐frequency words over pseudohomophones activated the right hippocampus and motor cortex. This could indicate that participants were perhaps trying to guess the right answer when recognising unfamiliar words based on the vocabulary entries in their memory. The hippocampus plays a role in word imageability (Klaver et al., 2005), which could explain why, unlike pseudohomophones, low‐frequency words activate the memory system by comparing the word representation to its meaning. It is possible that low‐frequency words and pseudohomophones activate phonological processing areas equally, and therefore, the corresponding areas did not show a differential activation.

Rolandic operculum, IFG, precentral gyrus, supramarginal gyrus, and the insula form part of the dorsal stream involved in translating sound into articulation (Saur et al., 2008; Tomasino et al., 2020) and their lesions were associated with phonological dyslexia (Tomasino et al., 2020). In word‐nonword recognition, they can act as a support to other phonological processing areas.

### 4.2 Psychopathology‐related traits and brain activity

This is the first study to have examined possible associations between the neural correlates of LDT and personality traits related to psychosis, psychopathy, and impulsivity. Of these traits, Motor Impulsivity was the trait related most consistently to lower activation in the brain areas that are typically involved in phonological processing and automatic word recognition (Saur et al., 2008; Steinbrink et al., 2009; Tomasino et al., 2020). Specifically, the right STG was less active when identifying low‐frequency words (over pseudohomophones), the left STG and cerebellum bilaterally were less active for real nonwords (over rest), the fusiform gyrus bilaterally and right pars triangularis of the IFG were less active when identifying words (over rest), and the left fusiform, right anterior insula, and right inferior temporal gyrus were less active when identifying real nonwords (over rest), in association with higher Motor Impulsivity.

As the left STG is strongly involved in phonological processing and found to be activated during the letter‐sound conversion (Simos et al., 2000), our results suggest some disruption of these functions in people with a relatively higher level of Motor Impulsivity (Lee et al., 2011). Some evidence suggests that the bilateral STG involvement is required for phonological processing of more difficult stimuli (Graves et al., 2008; Ramos Nuñez et al., 2020). This could be similar to people with aphasia or dyslexia who activate the right STG more strongly than controls as it may serve as a supporting structure and provide additional resources to cope with difficulties in phonological processing (Simos et al., 2000; Teki et al., 2013). It is also possible that people with higher impulsivity only superficially process unfamiliar words before reaching a decision and, thus, fail to strongly activate the STG bilaterally. Lastly, the right STG is also involved in successful inhibitory control (Horn et al., 2003) and this area is found to be structurally reduced in forensic samples with higher impulsivity (Müller et al., 2008).

Motor Impulsivity also modulated activity in the fusiform gyrus bilaterally, right IFG, and right insula, the areas known to be active in automatic recognition of lexical stimuli and selection from the competing lexical representations (Buchweitz et al., 2009; Fiez, 2001). The anterior insula bilaterally is involved in the auditory temporal processing, supports phonological representations of verbal stimuli, and is functionally connected with the left IFG (Steinbrink et al., 2009). Higher Motor Impulsivity was associated with lower activity in these areas in the right hemisphere. Therefore, this can indicate a reduced bilateral integration of the meaning and sound of mental lexical representations and selecting the appropriate outputs in those with higher impulsivity. Lastly, Motor Impulsivity was associated with lower cerebellar activity. The cerebellum has an important role in language, including word recognition (Mariën et al., 2013), and language proficiency (Baillieux et al., 2008; De Smet et al., 2013). The right cerebellum is also involved in phonological and semantic processing in reading (De Smet et al., 2013). It is also worth noting that the peak activity in none of these areas significantly correlated with LDT performance suggesting that these associations were not explained by any impulsivity‐related differences in performance. Another point worth noting is that native and non‐native English speakers had comparable LDT performance in this study, possibly due to all participants having studied at English‐speaking universities, and this may allowed the influence of Motor Impulsivity to emerge strongly across the entire sample.

Higher positive schizotypy (Unusual Experiences) was associated with lower activity in the left cerebellum when identifying low‐frequency words over real nonwords, similar to the activation observed in higher impulsivity during real nonword identification. The cerebellum is also functionally connected with frontal and temporal areas (Allen et al., 2005; Londei et al., 2009) that show aberrations during LDT in people with schizophrenia (Lam et al., 2012; Natsubori et al., 2014). The areas found associated with Motor Impulsivity in our study have also been found to be less active during phonological processing and automatic word recognition in people with psychosis (Li et al., 2010). Taken together, these observations suggest that positive schizotypy and impulsivity may share some variance associated with lower activation in areas functionally connecting phonological processing and lexical knowledge. The integration is important when identifying especially low‐frequency words as these, unlike real nonwords, activate appropriate vocabulary (lexical) entries in the memory.

Higher Meanness was uniquely associated with lower activity in the ventral diencephalon and caudate nucleus bilaterally when identifying nonwords (over words). This is in concordance with previous findings suggesting functional and structural impairments in the ventral striatum (Boccardi et al., 2013; Glenn & Yang, 2012) and caudate nucleus (Viding & McCrory, 2012) in higher psychopathy. Moreover, the ventral diencephalon as part of the striato‐thalamo‐frontal network was also found to show deficits in association with the antisocial traits in psychopathy (Hoppenbrouwers et al., 2013). Similarly, higher Boldness was associated with lower activity in the right posterior cingulate, an area previously found to be over‐activated in people with antisocial personality disorder, high psychopathy, and violent offending (Gregory et al., 2015). Overall, people with higher psychopathy trait scores showed lower neural activity when identifying nonwords over words, unlike the negative association found in positive schizotypy and Motor Impulsivity for words over nonwords. However, the psychopathy traits in healthy individuals were not directly associated with activations in areas specific to the response to lexical stimuli or reading, unlike in previous studies involving people with psychopathy and a history of violence (Kiehl et al., 2004; Montry et al., 2021). Further studies are necessary to clarify the exact mechanism through which psychopathy influences reading‐related skills.

### 4.3 Limitations

Participants included in this study had a relatively moderate range of scores on some schizotypal and psychopathic indices and this may have resulted in reduced power to examine the hypothesised relationships. A larger sample with a wider range of trait scores would be helpful to confirm and extend our findings.

### 4.4 Conclusions

This study examined neural activations during LDT in association with dimensional psychopathology‐related traits of positive schizotypy, psychopathy, and impulsivity. Our findings showing that higher Motor Impulsivity was strongly associated with lower activity in several areas strongly involved in word‐nonword recognition are in concordance with recent behavioural findings in an independent sample (Vanova et al., 2022). Higher positive schizotypal traits (Unusual Experiences) were associated with lower neural activity in the left cerebellum in low‐frequency words, an area with connection to lexical and phonological knowledge areas. Interestingly, Meanness and Boldness facets of psychopathy did not significantly associate with activity changes in any areas involved specifically involved in phonological processing or lexical representations despite previous studies indicating some potential anomalies in these reading skills at the behavioural and/or neural levels (Kiehl et al., 2004; Montry et al., 2021).

## CONFLICT OF INTEREST

The authors declare no competing interests.

## Supporting information


**Appendix S1** Supplementary tables

## Data Availability

The data used in this study are available from the corresponding author upon reasonable request.
