# Supplementary material for: Distinct neural signatures of schizotypy and psychopathy during visual word‐nonword recognition
Source: Hum Brain Mapp. 2022 Apr 18;43(12):3620–32. doi: 10.1002/hbm.25872 (PMC9294305; doi:10.1002/hbm.25872)
Supplement: Supplementary file 1 — Appendix S1 Supplementary tables [file HBM-43-3620-s001.docx]

**Supporting materials**

**Supplementary Table 1.** Sample characteristics.

| Variable/*N* | Men  (*N* = 7) | Women  (*N* = 15) | Overall sample  (*N* = 22) |
| --- | --- | --- | --- |
| **Age** (Mean [±*SD*]) | 27.14 (3.17) | 22.73 (.69) | 24.13 (5.40) |
| **Language** (Number [%]) |  |  |  |
| English | 1 (14.3%) | 10 (66.7%) | 11 (50%) |
| Other | 6 (85.7%) | 5 (33.3%) | 11 (50%) |
| **Ethnicity** (Number [%]) |  |  |  |
| White | 3 (42.9%) | 11 (73.3%) | 14 (63.6%) |
| Asian/Pacific Islander | 2 (28.6%) | 3 (20%) | 5 (22.7%) |
| Black/African American | 1 (14.3%) | 0 | 1 (4.5%) |
| Hispanic/Latino | 0 | 1 (6.7%) | 1 (4.5%) |
| Other | 1 (14.3%) | 0 | 1 (4.5%) |
| **Education** (Number [%]) |  |  |  |
| Higher Degree | 5 (71.4%) | 6 (40.0%) | 11 (50%) |
| First Degree | 1 (14.3%) | 8 (53.3%) | 9 (40.9%) |
| GCE A Level in 2+ | 1 (14.3%) | 1 (6.7%) | 2 (9.1%) |

**Supplementary Table 2.** Task-related activations (T-contrasts) across the entire sample (*n* = 22) [height threshold family wise error (FWE) corrected *p* < .05; extent threshold = 2 voxels].

|  |  |  |  | |  | | |  | | | |
| --- | --- | --- | --- | --- | --- | --- | --- | --- | --- | --- | --- |
| *Contrast* |  |  | Cluster level | | Peak level | | | MNI coordinates | | | |
| Area name | BA | Side | *P_FWE_* | *K_E_* | *P_FWE_* | *T* | | x y z (mm) | | | |
| ***Correct high-frequency words > rest*** | | | | | | | | | | | |
| Inferior occipital gyrus | 19 | Right | < .001 | 241 | < .001 | 9.78 | | 40 | | -82 | -4 |
|  | 18 |  |  |  | .004 | 7.86 | | 30 | | -88 | -4 |
| Postcentral gyrus | 3 | Left | < .001 | 983 | < .001 | 9.29 | | -46 | | -24 | 56 |
|  |  |  |  |  | < .001 | 9.27 | | -46 | | -22 | 42 |
|  |  |  |  |  | .003 | 8.07 | | -38 | | -30 | 48 |
| Fusiform gyrus | 37 | Left | < .001 | 476 | .001 | 8.86 | | -44 | | -60 | -20 |
| Inferior occipital gyrus |  |  |  |  | .003 | 8.02 | | -46 | | -64 | -10 |
| Middle temporal gyrus |  |  |  |  | .004 | 7.89 | | -46 | | -60 | -2 |
| Inferior occipital gyrus | 19 | Left | < .001 | 127 | .001 | 8.84 | | -36 | | -80 | -8 |
| Cerebellum | 37 | Right | < .001 | 360 | .003 | 8.06 | | 30 | | -42 | -26 |
| Culmen | 19 |  |  |  | .004 | 7.88 | | 10 | | -52 | -20 |
| Fusiform gyrus | 37 |  |  |  | .009 | 7.32 | | 42 | | -52 | -24 |
| Rolandic operculum | 48 | Left | .002 | 22 | .009 | 7.35 | | -46 | | -26 | 22 |
| Inferior frontal gyrus | 6 | Left | .001 | 28 | .014 | 7.08 | | -58 | | 10 | 26 |
| Insula | 48 | Left | .002 | 24 | .018 | 6.94 | | -44 | | 8 | 8 |
| Insula | 48 | Left | .011 | 7 | .019 | 6.89 | | -34 | | 8 | 10 |
| Insula | 48 | Left | .026 | 2 | .022 | 6.83 | | -32 | | 24 | 8 |
| Inferior frontal opercularis | 6 | Right | .010 | 8 | .023 | 6.78 | | 58 | | 12 | 18 |
| Postcentral gyrus | 4 | Right | .010 | 8 | .027 | 6.70 | | 56 | | -14 | 48 |
| Inferior temporal gyrus | 37 | Right | .026 | 2 | .034 | 6.56 | | 42 | | -62 | -6 |
| ***Correct low-frequency words > rest*** | | | | | | | | | | | |
| Rolandic operculum | 48 | Left | < .001 | 1669 | < .001 | 12.64 | | -42 | | 6 | 16 |
| Insula |  |  |  |  | < .001 | 9.65 | | -34 | | 0 | 14 |
| Putamen |  |  |  |  | < .001 | 9.25 | | -26 | | 6 | -8 |
| Postcentral gyrus | 3 | Left | < .001 | 2660 | < .001 | 11.80 | | -38 | | -32 | 52 |
| Supplementary motor area | 6 |  |  |  | < .001 | 10.71 | | -8 | | 0 | 72 |
| Postcentral gyrus |  |  |  |  | < .001 | 10.43 | | -46 | | -28 | 58 |
| Inferior occipital gyrus | 19 | Left | < .001 | 2837 | < .001 | 10.68 | | -34 | | -82 | -8 |
| Middle temporal gyrus | 37 |  |  |  | < .001 | 10.63 | | -46 | | -60 | -2 |
| Inferior occipital gyrus |  |  |  |  | < .001 | 10.42 | | -46 | | -62 | -10 |
| Precentral gyrus | 6 | Right | < .001 | 70 | .001 | 8.64 | | 58 | | 10 | 28 |
| Inferior frontal opercularis | 44 |  |  |  | .028 | 6.79 | | 48 | | 12 | 28 |
| Thalamus |  | Right | < .001 | 82 | .004 | 7.97 | | 12 | | -14 | 12 |
| Ventral anterior nucleus |  |  |  |  | .007 | 7.60 | | 14 | | 6 | 10 |
|  |  |  |  |  | .008 | 7.50 | | 14 | | -6 | 10 |
| Postcentral gyrus | 3 | Right | < .001 | 92 | .005 | 7.79 | | 38 | | -34 | 50 |
| Supramarginal gyrus | 40 |  |  |  | .023 | 6.90 | | 36 | | -34 | 42 |
| Postcentral gyrus | 48 | Left | .012 | 5 | .007 | 7.64 | | -48 | | -8 | 18 |
| Insula | 48 | Right | < .001 | 35 | .009 | 7.45 | | 36 | | 4 | 10 |
| Postcentral gyrus | 3 | Right | .008 | 7 | .018 | 7.06 | | 36 | | -24 | 38 |
|  |  |  |  |  |  |  | |  | |  |  |
| ***Correct real nonwords > rest*** | | | | | | | | | | | |
| Postcentral gyrus | 3 | Left | < .001 | 1360 | < .001 | 11.52 | | | -58 | -12 | 40 |
| Precentral gyrus | 43 |  |  |  | < .001 | 11.04 | | | -58 | -10 | 22 |
| Inferior parietal gyrus | 4 |  |  |  | .001 | 8.91 | | | -48 | -20 | 42 |
| Inferior temporal gyrus | 37 | Left | < .001 | 915 | < .001 | 11.03 | | | -52 | -64 | -6 |
| Inferior occipital gyrus | 19 |  |  |  | < .001 | 9.90 | -36 | | | -82 | -6 |
| Middle temporal gyrus | 37 |  |  |  | < .001 | 9.51 | -44 | | | -58 | -4 |
| Inferior occipital gyrus | 19 | Right | < .001 | 495 | < .001 | 10.24 | 42 | | | -78 | -8 |
|  |  |  |  |  | 0.001 | 8.77 | 36 | | | -86 | -6 |
| Inferior temporal gyrus | 37 |  |  |  | 0.012 | 7.22 | 42 | | | -64 | -8 |
| Rolandic operculum | 48 | Left | < .001 | 588 | < .001 | 10.23 | -40 | | | 6 | 16 |
| Insula |  |  |  |  | < .001 | 9.35 | -44 | | | 4 | 8 |
| Precentral gyrus | 44 |  |  |  | .001 | 9.06 | -56 | | | 10 | 28 |
| Fusiform gyrus | 37 | Right | < .001 | 383 | .001 | 8.86 | 14 | | | -48 | -20 |
|  |  |  |  |  | .002 | 8.28 | 26 | | | -42 | -26 |
|  |  |  |  |  | .003 | 8.00 | 28 | | | -34 | -26 |
| Inferior frontal opercularis | 48 | Right | < .001 | 180 | .001 | 8.76 | 52 | | | 14 | 28 |
|  | 44 |  |  |  | .031 | 6.65 | 56 | | | 14 | 12 |
| Supplementary motor area | 6 | Left | < .001 | 381 | .002 | 8.41 | -4 | | | 4 | 52 |
|  |  |  |  |  | .002 | 8.19 | -6 | | | 0 | 60 |
| Middle cingulum | 32 | Right |  |  | .004 | 7.91 | 6 | | | 26 | 42 |
| Insula | 48 | Left | .004 | 15 | .019 | 6.93 | -34 | | | 24 | 12 |
| Thalamus (Ventral lateral nucleus) |  | Left | .004 | 15 | .021 | 6.88 | -18 | | | -14 | 4 |
| Inferior frontal triangularis | 48 | Right | .025 | 2 | .029 | 6.69 | 38 | | | 24 | 12 |
|  |  |  |  |  |  |  |  | | |  |  |
| ***Correct pseudohomophones > rest*** | | | | | | | | | | | |
| Postcentral gyrus | 2 | Left | < .001 | 1065 | < .001 | 10.75 | | | -42 | -28 | 46 |
| Precentral gyrus | 3 |  |  |  | < .001 | 9.82 | | | -44 | -30 | 54 |
| Inferior parietal gyrus | 43 |  |  |  | .002 | 8.24 | | | -58 | -10 | 24 |
| Inferior temporal gyrus | 37 | Left | < .001 | 684 | < .001 | 9.98 | -48 | | | -58 | -4 |
| Middle temporal gyrus |  |  |  |  | .001 | 8.69 | -48 | | | -56 | -12 |
| Fusiform gyrus |  |  |  |  | .002 | 8.31 | -44 | | | -60 | -20 |
| Rolandic operculum | 48 | Left | < .001 | 367 | < .001 | 9.67 | -40 | | | 6 | 16 |
| Inferior frontal gyrus | 44 |  |  |  | .001 | 8.85 | -48 | | | 12 | 20 |
| Inferior frontal gyrus |  |  |  |  | .004 | 7.77 | -54 | | | 10 | 28 |
| Insula | 48 | Left | < .001 | 95 | .001 | 8.65 | -34 | | | 26 | 8 |
| Inferior frontal gyrus | 45 |  |  |  | .037 | 6.50 | -42 | | | 22 | 14 |
| Supplementary motor area | 6 | Left | < .001 | 110 | .007 | 7.51 | -2 | | | 6 | 54 |
|  | 32 |  |  |  | .016 | 6.98 | -6 | | | 14 | 46 |
| Fusiform gyrus | 37 | Right | < .001 | 40 | .008 | 7.41 | 38 | | | -50 | -20 |
| Inferior frontal gyrus | 48 | Right | .003 | 17 | .009 | 7.32 | 36 | | | 28 | 10 |
| Inferior frontal opercularis | 44 | Right | .004 | 16 | .011 | 7.18 | 52 | | | 14 | 28 |
| Inferior occipital gyrus | 19 | Right | .005 | 14 | .012 | 7.16 | 40 | | | -82 | -2 |
| Fusiform gyrus | 37 | Right | .001 | 30 | .013 | 7.09 | 34 | | | -36 | -26 |
| Supplementary motor area | 32 | Right | .008 | 10 | .019 | 6.88 | 4 | | | 24 | 44 |
| Inferior occipital gyrus | 19 | Left | .010 | 8 | .029 | 6.64 | -38 | | | -76 | -8 |
| Superior frontal gyrus | 6 | Left | .026 | 2 | .033 | 6.57 | -26 | | | -6 | 68 |
|  |  |  |  |  |  |  |  | | |  |  |
| ***Incorrect real nonwords > rest (n = 18)*** | | | | | | | | | | | |
| Supplementary motor area | 32 | Right | < .001 | 352 | .001 | 10.19 | 6 | | | 30 | 34 |
|  | 6 |  |  |  | .003 | 9.52 | 2 | | | 12 | 58 |
|  |  |  |  |  | .004 | 9.26 | 0 | | | 16 | 50 |
| Insula | 47 | Left | < .001 | 139 | .002 | 9.65 | -36 | | | 26 | 0 |
| Supplementary motor area | 6 | Left | .002 | 9 | .017 | 8.09 | -8 | | | 0 | 64 |
|  |  |  |  |  |  |  |  | | |  |  |
| ***Incorrect pseudohomophones > rest (n = 18)*** | | | | | | | | | | | |
| Supplementary motor area | 8 | Left | <.001 | 322 | <.001 | 11.68 | -2 | | | 22 | 46 |
|  | 6 |  |  |  | .008 | 8.38 | -6 | | | 10 | 56 |
| Middle cingulum | 32 | Right |  |  | .013 | 8.03 | 6 | | | 30 | 34 |
| Postcentral gyrus | 2 | Left | <.001 | 65 | .001 | 9.75 | -42 | | | -30 | 44 |
|  | 40 |  |  |  | .012 | 8.09 | -30 | | | -36 | 46 |
| Inferior frontal triangularis | 48 | Left | .011 | 5 | .025 | 7.52 | -50 | | | 16 | 18 |
| Inferior frontal opercularis | 44 | Left | .011 | 5 | .042 | 7.14 | -54 | | | 12 | 22 |
|  |  |  |  |  |  |  |  | | |  |  |
| ***Correct high-frequency words > correct real nonwords*** | | | | | | | | | | | |
| Angular gyrus | 39 | Left | .001 | 27 | .005 | 7.76 | -44 | | | -70 | 30 |
|  |  |  |  |  |  |  |  | | |  |  |
| ***Correct high-frequency words > correct pseudohomophones*** | | | | | | | | | | | |
| Precuneus | 5 | Right | < .001 | 883 | .001 | 8.68 | 4 | | | -54 | 60 |
| Middle cingulate | 23 |  |  |  | .002 | 8.28 | 6 | | | -22 | 42 |
|  |  |  |  |  | .003 | 8.15 | 2 | | | -48 | 50 |
| Angular gyrus | 39 | Left | < .001 | 228 | .002 | 8.19 | -44 | | | -66 | 32 |
|  |  |  |  |  | .004 | 7.93 | -44 | | | -60 | 26 |
| Superior temporal gyrus | 40 | Right | < .001 | 34 | .008 | 7.49 | 60 | | | -42 | 24 |
| Sup. medio-frontal gyrus | 10 | Right | < .001 | 103 | .009 | 7.43 | 10 | | | 54 | 4 |
| Medio-frontal orbital gyrus | 11 |  |  |  | .015 | 7.09 | 14 | | | 56 | -4 |
| Paracentral lobule |  | Right | .013 | 5 | .013 | 7.18 | 16 | | | -28 | 60 |
| Angular gyrus | 39 | Right | < .001 | 78 | .023 | 6.83 | 46 | | | -54 | 34 |
|  |  |  |  |  | .033 | 6.63 | 50 | | | -58 | 26 |
|  |  |  |  |  |  |  |  | | |  |  |
| ***Correct low-frequency words > correct high-frequency words*** | | | | | | | | | | | |
| Inferior frontal triangularis | 47 | Left | .009 | 7 | .004 | 7.87 | -30 | | | 32 | -2 |
| Inferior temporal gyrus | 20 | Left | .008 | 8 | .015 | 7.12 | -46 | | | -44 | -14 |
|  |  |  |  |  |  |  |  | | |  |  |
| ***Correct low-frequency words > correct pseudohomophones*** | | | | | | | | | | | |
| Precentral gyrus | 6 | Right | < .001 | 45 | .008 | 7.45 | 18 | | | -22 | 68 |
| Hippocampus | 20 | Right | .003 | 16 | .008 | 7.40 | 36 | | | -22 | -12 |
|  |  |  |  |  |  |  |  | | |  |  |
| ***Correct pseudohomophones > incorrect pseudohomophones (n = 18)*** | | | | | | | | | | | |
| Precentral gyrus | 4 | Left | .001 | 21 | .015 | 8.03 | -34 | | | -28 | 64 |
|  | 6 |  |  |  | .016 | 7.99 | -32 | | | -20 | 68 |
|  |  |  |  |  |  |  |  | | |  |  |
| ***Incorrect real nonwords > correct real nonwords (n = 18)*** | | | | | | | | | | | |
| Insula | 47 | Left | < .001 | 30 | .005 | 9.05 | -36 | | | 22 | -2 |
| Middle cingulum | 32 | Right | .002 | 8 | .025 | 7.88 | 6 | | | 30 | 32 |
|  |  |  |  |  |  |  |  | | |  |  |
| ***Rest > correct real nonwords*** | | | | | | | | | | | |
| Medial superior frontal gyrus |  | Left | <.001 | 41 |  | 7.49 | -2 | | | 58 | 16 |
| Angular gyrus |  |  | .020 | 3 |  | 6.55 | -40 | | | -62 | 44 |
| Angular gyrus |  |  | .017 | 4 |  | 6.54 | -44 | | | -68 | 32 |
|  |  |  |  |  |  |  |  | | |  |  |
| ***Rest > correct pseudohomophones*** | | | | | | | | | | | |
| Middle temporal gyrus |  | Left | < .001 | 110 | < .001 | 9.94 | -54 | | | 10 | -26 |
|  |  |  |  |  | .002 | 8.20 | -62 | | | -8 | -22 |
|  |  |  |  |  | .004 | 7.81 | -58 | | | 2 | -22 |
| Medial superior frontal gyrus |  | Right | < .001 | 412 | .001 | 8.83 | 4 | | | 60 | 10 |
|  |  |  |  |  | .015 | 7.03 | 8 | | | 54 | -4 |
|  |  | Left |  |  | .023 | 6.77 | -2 | | | 52 | 22 |
| Angular gyrus |  | Left | < .001 | 149 | .001 | 8.79 | -42 | | | -68 | 34 |
|  |  |  |  |  | .006 | 7.56 | -50 | | | -60 | 34 |
| Cuneus |  | Right | .005 | 13 | .004 | 7.83 | 12 | | | -90 | 14 |
| Middle temporal gyrus |  | Right | .001 | 28 | .009 | 7.35 | 64 | | | -12 | -16 |
| Posterior cingulate |  | Left | < .001 | 113 | .018 | 6.93 | -8 | | | -42 | 36 |
| Anterior cingulate |  | Right | .001 | 27 | .019 | 6.89 | 8 | | | 36 | -8 |
| Superior frontal gyrus |  | Left | .022 | 3 | .023 | 6.78 | -14 | | | 64 | 12 |
| Angular gyrus |  | Right | .008 | 10 | .028 | 6.65 | 50 | | | -60 | 34 |
| Anterior cingulate |  | Right | .026 | 2 | .041 | 6.44 | 4 | | | 26 | -14 |
| Gyrus rectus |  | Right | .022 | 3 | .042 | 6.42 | 2 | | | 28 | -24 |
|  |  |  |  |  |  |  |  | | |  |  |

*Note.* BA = Brodmann area; FWE = Family-wise Error; uncor. = uncorrected; MNI = Montreal Neurological Institute coordinate system; O-LIFE = Oxford-Liverpool Inventory of Feelings and Experiences; TriPM = Triarchic Psychopathy Measure; BIS-11 = Barratt Impulsiveness Sc

**Supplementary Table 3.** Correlations between participant-specific activation contrast image values (for peaks negatively associated with psychopathology-related traits) and LDT performance variables.

| *Contrast*  Area of activation (BA equiv.) | Correct high-frequency | Correct low-frequency | Correct pseudo-homophones | | Correct real nonwords | Correct high-frequency RT | Correct low-frequency RT | | Correct pseudo-homophones RT | | Correct real nonwords RT |
| --- | --- | --- | --- | --- | --- | --- | --- | --- | --- | --- | --- |
|  | *r* (*p*) | *r* (*p*) | *r* (*p*) | *r* (*p*) | | *r* (*p*) | | *r* (*p*) | | *r* (*p*) | *r* (*p*) |
| ***BIS-11 Motor*** | | | | | | | | | | | |
| *Correct high-frequency > rest*  Left fusiform gyrus (BA 37) | .168 (.456) |  |  |  | | .046 (.841) | |  | |  |  |
|  |  |  |  |  | |  |  |  | |  |  |
| *Correct high-frequency > rest*  Left superior temporal gyrus (BA 22) | -.067 (.766) |  |  |  | | .104 (.644) | |  | |  |  |
|  |  |  |  |  | |  |  |  | |  |  |
| *Correct high-frequency > rest*  Right inferior frontal triangularis (BA 45) | .075 (.742) |  |  |  | | .100 (.657) | |  | |  |  |
|  |  |  |  |  | |  |  |  | |  |  |
| *Correct low-frequency > rest* |  | .265 (.233) |  |  | |  | | -.035 (.879) | |  |  |
| Left fusiform gyrus (BA 37) |  |  |  |  | |  | |  |  |  |  |
| *Correct low-frequency > rest*  Right fusiform gyrus (BA 42) |  | .324 (.141) |  |  | |  | | .103 (.649) | |  |  |
|  |  |  |  |  | |  | |  |  |  |  |
|  |  |  |  |  | |  | |  |  |  |  |
| *Correct low-frequency > rest*  Right inferior frontal triangularis (BA 45) |  | .156 (.488) |  |  | |  | | .281 (.206) | |  |  |
|  |  |  |  |  | |  | |  |  |  |  |
| *Correct low-frequency > rest*  Right inferior temporal gyrus (BA 37) |  | -.101 (.656) |  |  | |  | | .103 (.648) | |  |  |
| *Correct real nonwords > rest*  Right inferior temporal gyrus (BA 36) |  |  |  | .393 (.071) | |  | |  | |  | .114 (.614) |
|  |  |  |  |  |  |  | |  | |  |  |
| *Correct real nonwords > rest*  Left fusiform gyrus (BA 37) |  |  |  | .253 (.256) | |  | |  | |  | .032 (.886) |
|  |  |  |  |  |  |  | |  | |  |  |
| *Correct real nonwords > rest*  Right Cerebellum |  |  |  | .101 (.654) | |  | |  | |  | -.061 (.788) |
| *Contrast*  Area of activation (BA equiv.) | Correct high-frequency | Correct low- frequency | Correct pseudo-homophones | Correct real nonwords | | Correct high- frequency RT | | Correct low- frequency RT | | Correct pseudo-homophones RT | Correct real nonwords RT |
|  | *r* (*p*) | *r* (*p*) | *r* (*p*) | *r* (*p*) | | *r* (*p*) | | *r* (*p*) | | *r* (*p*) | *r* (*p*) |
| *Correct real nonwords > rest*  Right inferior frontal triangularis (BA 47) |  |  |  | .275 (.215) | |  | |  | |  | .146 (.518) |
|  |  |  |  |  |  |  | |  | |  |  |
| *Correct real nonwords > rest*  Right superior temporal gyrus (BA 48) |  |  |  | .115 (.610) | |  | |  | |  | .011 (.962) |
| *Correct real nonwords > rest*  Right postcentral gyrus (BA 3) |  |  |  | .093 (.682) | |  | |  | |  | .167 (.457) |
|  |  |  |  |  |  |  | |  | |  |  |
|  |  |  |  |  |  |  | |  | |  |  |
|  |  |  |  |  |  |  | |  | |  |  |
| *Correct low-frequency > correct pseudo.*  Right superior temporal gyrus (BA 22) |  | .145 (.520) | -.100 (.657) |  | |  | | .212 (.344) | | .183 (.415) |  |
| *Correct real nonwords – correct pseudo* |  |  | -.052 (.817) | .186 (.407) | |  | |  | | .077 (734) | .048 (.831) |
| Left Cerebellum |  |  |  |  |  |  | |  | |  |  |
| ***O-LIFE Unusual Experiences*** | | | | | | | | | | | |
| *Correct low-frequency > correct real nonw.*  Left Cerebellum |  | .030 (.895) |  | .244 (.273) | |  | | .235 (.293) | |  | .218 (.331) |
| ***TriPM - Boldness*** | | | | | | | | | | | |
| *Correct correct pseudo. > low-frequency* |  | -.163 (.468) | .289 (.192) |  | |  | |  | | .120 (.595) | .242 (.278) |
| Right Posterior Cingulate |  |  |  |  | |  | |  | |  |  |
| ***Tri-PM Meanness*** | | | | | | | | | | | |
| *Correct correct real nonw. > high-frequency*  Left ventral diencephalon (BA 25) | .141 (.530) |  |  | -.043 (.849) | | .204 (.362) | |  | |  | .133 (.556) |
|  |  |  |  |  | |  | |  | |  |  |

*Note:* BA = Brodmann area
